# Experimental Study of Ghost Imaging in Underwater Environment

**DOI:** 10.3390/s22228951

**Published:** 2022-11-18

**Authors:** Heng Wu, Ziyan Chen, Chunhua He, Lianglun Cheng, Shaojuan Luo

**Affiliations:** 1 Guangdong Provincial Key Laboratory of Cyber-Physical System, School of Automation, Guangdong University of Technology, Guangzhou 510006, China; 2 School of Computer, Guangdong University of Technology, Guangzhou 510006, China; 3 School of Chemical Engineering and Light Industry, Guangdong University of Technology, Guangzhou 510006, China

**Keywords:** underwater ghost imaging, compressed sensing, underwater imaging, single-pixel imaging

## Abstract

Underwater imaging technique is a crucial tool for humans to develop, utilize, and protect the ocean. We comprehensively compare the imaging performance of twenty-four ghost imaging (GI) methods in the underwater environment. The GI methods are divided into two types according to the illumination patterns, the random and orthogonal patterns. Three-group simulations were designed to show the imaging performance of the twenty-four GI methods. Moreover, an experimental system was built, and three-group experiments were implemented. The numerical and experimental results demonstrate that the orthogonal pattern-based compressed sensing GI methods have strong antinoise capability and can restore clear images for underwater objects with a low measurement number. The investigation results are helpful for the practical applications of the underwater GI.

## 1. Introduction

Underwater imaging (UI) technique plays a significant role in the underwater target observation, detection, seabed resource exploration, environmental monitoring, and so on [1,2,3]. However, traditional imaging techniques encounter many problems in the underwater environment, such as the noise contamination, low image resolution, short imaging distance, etc. These are mainly caused by the light absorption of water and the scattering effects of suspended microparticles. Many methods have been developed to improve the quality of underwater images over the past few years, such as the metalens-assisted system [4], polarization filter and histogram attenuation prior [5], crosstalk compensation [6], active polarized illumination and average filtering technology [7], learning-based methods [8,9], etc. Recently, ghost imaging (GI) has been employed in UI [10,11]. Different from the conventional UI methods, GI uses the second-order correlation to recover an object image [12,13,14,15,16,17,18]. Compared with the conventional UI technologies, GI has many advantages in the underwater environment [19,20,21,22,23,24,25,26,27,28,29], such as imaging in scattering medium [19,20,21,22,23], in turbid media [24,25,26], in low light environment [27], and imaging with multiple spectrums [28,29].

Underwater GI (UGI) has attracted great attention, and many UGI methods have been developed recently. For example, Zhang et al. studied the effects of seawater on the quality of GI [30]. The results indicated that GI could achieve better imaging quality than conventional imaging. In addition, the compressed sensing (CS) could further enhance the visibility of ghost images with fewer measurements. Gao et al. investigated GI in transparent liquid and found that increasing the liquid refractive index can raise the imaging resolution [31]. Luo et al. reported an underwater computational GI (CGI) scheme with shaped Lorentz sources [32]. The results show that the long-distance underwater CGI quality can be enhanced. Wang et al. demonstrated the influence of uneven temperature distribution on imaging quality of CGI in the underwater environment [33]. They found that imaging quality presents an improvement trend as the water temperature increases. Additionally, the Push-Broom- [34], wavelet enhancement- [35] and deep learning-based [36] methods have also been used to improve the quality of UGI. Unlike [30,31,32,33,34,35,36], some works especially focused on the influence of the water turbidity on the CGI. Le et al. presented a CGI method in the underwater conditions [37]. They investigated the image quality of CGI in different turbidities and from different angles. The results are rather desirable. Bina et al. proposed the backscattering differential GI scheme in turbid media [24]. The results demonstrated that the proposed method recovers images with a contrast better than standard noncorrelated direct imaging. Liu et al. studied the influence of turbid media at different locations in CGI [25]. They found that the scattering medium in the illumination path could decrease the image quality, while it had almost no effect if it were only in the detection path. Yuan et al. designed a method that enables GI to noninvasively image objects through turbid media, and the method did not have a size limitation for the object [26]. The underwater turbulence can make the suspended microparticles move, which changes the directions of the scattering lights, resulting the image degradation of UGI. To address this problem, Luo et al. developed an imaging formula for the CGI operating in the oceanic turbulence [38]. They found that the propagation distance had a great influence on the image quality. Zhang et al. built a physical model of GI through oceanic turbulence and obtained theoretical expressions for the visibility of GI in oceanic turbulence [39]. The results indicated that the quality of GI is related to the turbulence intensity and light propagation distance. In UGI, the phase of light sources has also influenced the image quality. Liu et al. reported a UGI scheme with a partially coherent beam carrying twist phase (twisted Gaussian Schell-model beam) in the presence of oceanic turbulence [40]. The numerical results showed that the image quality of the proposed method can be maintained at an acceptable level. In contrast to the numerical study in [40], Yin et al. experimentally investigated the imaging quality of GI in the underwater environment [41]. The results indicated that GI had the turbulence-free ability in the environment of the low temperature gradient, water vibration, and turbid media. Wu et al. also experimentally studied the antidisturbance ability of UGI [42]. The ultrasonic waves are utilized to create the water disturbance. The experimental results reflected that the image quality of GI is better than that of classic imaging method in the underwater disturbance environment. The abovementioned works demonstrate that GI is a very promising technique in the UI area and can be considered as an alternative scheme of classical UI. However, in GI, the high image quality usually requires many measurements, which limits the practical applications of GI.

In this paper, we comprehensively study the imaging performance of twenty-four GI methods in the underwater environment by numerical and practical experiments. We create a unified UGI model and analyze the image reconstruction theory of GI methods. The GI methods are divided into two groups based on the illumination patterns, and only the imaging effect in low measurement number condition is investigated. In the simulations, the underwater noise is supposed to be the white gaussian noise. In the actual experiments, an experimental setup is constructed to simulate the underwater environment. Three-group numerical and actual experiments are respectively implemented to check the imaging effect. The effectiveness and performance of the GI methods are verified and analyzed.

## 2. Method

### 2.1. Underwater Ghost Imaging Model

**Figure 1:**
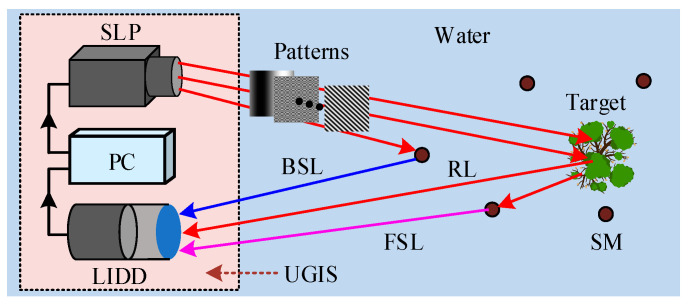
Schematic diagram of the UGIS model, RL: reflective light. The PC first controls the SLP to send structured illumination patterns onto the target, then controls the LIDD to record the light intensity, and finally restores the target image. The noises in this model mainly come from the BSL and FSL.

Figure 1 displays the schematic diagram of an UGI system (UGIS) model. The UGIS includes three parts, a structured light projector (SLP), light intensity detection device (LIDD), and a personal computer (PC). The SLP projects structured illumination patterns $P_{n}(x,y)$ onto the target. Here, the pattern can be the random pattern [14,15], Hadamard pattern [21,35], Fourier pattern [43,44], etc. The LIDD records the corresponding light intensity value $I_{n}$. The PC controls the pattern sending and light intensity recording. Note that the SLP is composed of a projective lens and light modulation device (e.g., digital micromirror device, spatial light modulator). The LIDD is made up of a bucket detector and a collective lens. As shown in Figure 1, the light intensity value $I_{n}$ is written as follows [13,14,45]:

(1) $$I_{n}=\iint P_{n}(x,y)T(x,y)dxdy+noise_{n}$$

where T(x,y) is the target function, and N and (x,y) are respectively the total pattern number and pixel coordinate, n=1,2,⋯,N. Here, the noise $noise_{n}$ mainly contains two parts, the back scattering light (BSL) and forward scattering light (FSL), which are produced by the suspended microparticles (SM). In Equation (1), the underwater noise $noise_{n}$ is the major factor that deteriorates the image quality.

Many methods have been developed to reconstruct the ghost images from the patterns $P_{n}(x,y)$ and light intensity values $I_{n}$, including the correlation calculation, compressive sensing, pseudo-inverse matrix, Fourier spectrum acquisition, deep learning methods, and so on.

### 2.2. GI Image Reconstruction

Correlation calculation (COC). The COC is the main image restoration method in the GI area. The target image T(x,y) restored by COC is given by [13,14,15,16,17,44]:

(2) T(x,y)=〈PI〉−〈P〉〈I〉

where $P=[P_{1}(x,y),P_{2}(x,y),\cdots ,P_{N}(x,y)]$ denotes the pattern sequence, $I=\left[I_{1},I_{2},\cdots ,I_{N}\right]$ is the corresponding light intensity sequence (LIS), PI is an element-by-element product, and the bracket 〈〉 is a function that is used to calculate the mean value of the input variable and is defined as $\langle \cdot \rangle =\frac{1}{N}\sum_{n=1}^{N}\cdot$.

Following the COC, many improved GI image reconstruction methods have been proposed, such as the differential GI (DGI) [46], normalized GI (NGI) [47], “Russian dolls” GI (RD) [48], 4-connected-region-based CGI (CR) [49], low-rank minimization GI (LGI) [50], and zigzag scanning-based online adaptive CGI (ZzGI) [51]. Compared with the COC in Equation (2), [46,47,48,49,50,51] can further enhance the GI image quality.

Compressive sensing (CS). CS has the advantages of recovering signals with high quality in sub-Nyquist conditions. Thus, CS has been widely used in GI to reduce measurements and improve the imaging performance in the past few years [52,53,54]. Unlike the COC-based GI methods, CS GI can reconstruct the object image with more details and higher contrast [52,55,56,57,58]. The mathematical model of CS GI is usually expressed as

(3) I=AX+b

where X is the object vector, and A denotes the measurement matrix,

(4) $$A={\left[P_{1},P_{2},\cdots ,\begin{array}{c} P_{n}, \end{array}\cdots ,P_{N}\right]}^{T}$$

where $P_{n}$ is a column vector, b denotes the noise vector, and T is the matrix transposition. Note that reshape() is a MATLAB function and $reshape(P_{n}(x,y),M,1)$ means to change the pattern matrix $P_{n}(x,y)$ into a column vector M×1. Here, M is the total pixel number of the pattern matrix $P_{n}(x,y)$. The orthogonal matching pursuit (OMP) [52] and total variation augmented Lagrangian alternating direction algorithm (TVAL3) method [53] can be used for the GI image reconstruction in Equation (3). Here, the GI with OMP and TVAL3 are written as OGI [52] and TV [53], respectively. Many CS GI methods have been developed, such as CS wavelet enhancement GI (WGI) [35],”Cake-Cutting” GI (CC) [55], total variation regularization prior-based GI (TR) [56,57], sparse representation prior-based GI (SPGI) [57], and point spread function-based GI (PSF) [58].

### 2.3. Image Reconstruction with Other Methods

Fourier spectrum acquisition. In 2015, Zhang et al. reported a single-pixel imaging (SPI) scheme by acquiring the Fourier spectrum (FSPI) [43]. FSPI uses the four-step phase-shifting sinusoid patterns for the structured illumination. Since this method utilizes the positive and inverse Fourier transform to restore images, the noise term can be removed [42,43]. The imaging system of FSPI can also be used for GI. The main difference between FSPI and GI lies in the image reconstruction algorithm.

Pseudo-inverse matrix (PIM). As for the mathematical model in Equation (3), the object image $X_{0}$ can be recovered by the pseudo-inverse matrix, $X_{0}=PI$, where P is the PIM of A. Gong [59], Czajkowski et al. [60], and Pastuszczak et al. [61] have respectively developed three PIM-based image restoration methods that can be used for GI. The abbreviations of the three methods are the pseudo-inverse GI (PGI) [59], Fourier domain regularized inversion (DRI) [60], and differential DRI (DDRI) [61], respectively.

Except for the abovementioned methods, there are many special image reconstruction schemes for GI, such as the correspondence imaging (CI) [62], preconditioned deconvolution GI (PreGI) [63], alternating projection GI (APGI) [64], scalar-matrix-structured GI (SMGI) [65], fast Walsh–Hadamard transform (FWHT) [66], truncated singular-valued decomposition-based GI (TSGI) [67], and deep learning GI [68,69,70,71].

## 3. Results

### 3.1. Simulation Results and Analysis

**Figure 2:**
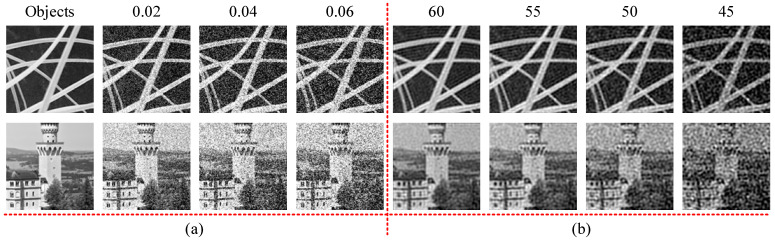
(**a**) Objects for simulations and simulated images with WGN at variances of 0.02, 0.04, and 0.06. (**b**) Images restored by FSPI at the noise levels of 60, 55, 50 and 45.

In the simulations, twenty-four GI methods are used for comparison. The tool for simulation and numerical calculations is MATLAB R2022a. Table 1 shows the pattern types of the twenty-four GI methods. Here, the SPI methods, such as the FSPI, DRI, DDRI, etc., are implemented in a GI model. Therefore, the SPI methods are also called GI methods. Note that the pattern type here is the same as the one in the original paper. We assume that the underwater noise is white gaussian noise (WGN) and three-group simulations are conducted. The WGN level of each group is shown in Table 2. The patterns in the three-group simulations are complete the same. The difference is adding WGN or not. The noise levels of WGN are also different. Note that a smaller noise level means more noises in the signal, and the noise level refers to the signal-to-noise ratio (*SNR*). We use the MATLAB function awgn (*x*, *SNR*, *signalpower*) to add WGN into the input signal, where *x*, *SNR* and *signalpower* are the input signal, noise level, and signal power type, respectively. The *signalpower* is specified as “measured” in all the simulations. Two objects (128 × 128 pixels) are used for simulations, as shown in Figure 2a. Additionally, Figure 2a also presents the simulated images that are contaminated by the WGN. Here, the WGN is added into the two object images by the MATLAB function imnoise () with variances of 0.02, 0.04, and 0.06, respectively. Figure 2b shows the simulated GI results recovered by FSPI with four noise levels (60, 55, 50, and 45), where the noises are added by the awgn () function. As shown in Figure 2, when the noise levels of 60 and 55 are chosen, it can be found that the difference between the original object images and reconstructed images with WGN is small. However, when the noise levels of 55 and 45 are used, the difference is dramatic. Consequently, the close noise levels of 50 and 45 are used for simulations.

The measurement number for GI with Fourier patterns is 3274, and for GI with random, discrete cosine and orthogonal patterns, it is 3276. The reason is that some patterns are dropped during the generation of Fourier patterns [43,44].

#### 3.1.1. Results without WGN

**Figure 3:**
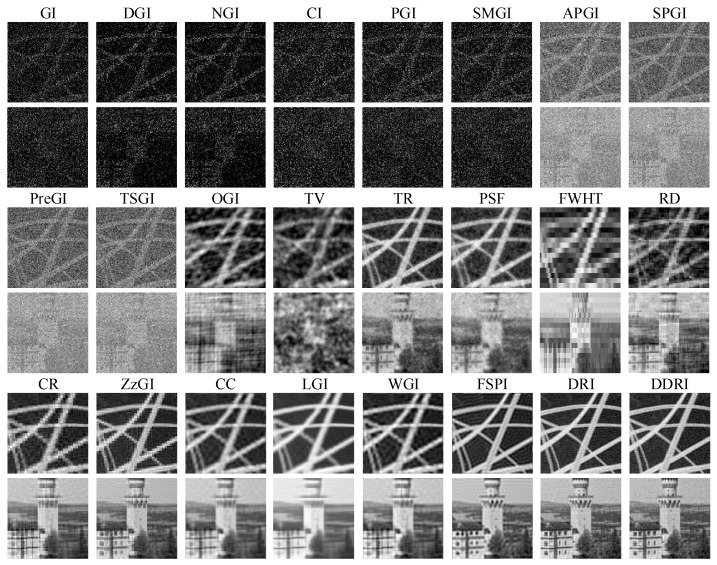
Simulation results of twenty-four methods without WGN. The results are obtained in ideal environment (without any noises).

Figure 3 shows the simulation results of twenty-four methods without WGN. For the random patterns, the CS GI methods, such as OGI, TV, TR, and PSF, have better imaging performance than the other methods (e.g., GI, DGI, TSGI, etc.). In the four CS GI methods, the visual effect of TR and PSF is better than that of OGI and TV. Except for FWHT and CR, the other orthogonal pattern GI methods (e.g., CR, DRI, DDRI, etc.) can obtain good imaging effect, especially for the orthogonal pattern CS GI methods (e.g., CC and WGI). The FSPI is superior to the FWHT, CR, and all the random pattern-based GI methods. The peak signal-to-noise ratio (PSNR) and root mean square error (RMSE) are used to evaluate the quality of the images in Figure 3. The definitions of PSNR and RMSE are detailed in [49].

Table 3 presents the quantitatively evaluation results corresponding to Figure 3. Note that the PSNR and RMSE of images with bad visual effect are not calculated, e.g., GI, DGI, NGI, etc. The DRI that has the highest PSNR and lowest RMSE achieves the best imaging performance. From the Figure 3 and Table 3, the imaging performance of GI methods with random patterns is commonly poor in low measurement number conditions. However, with the help of CS technique, the imaging effect can be improved. The Fourier, discrete cosine (DCT), and other orthogonal pattern GI methods can obtain high quality images, which are better than the random pattern GI methods.

#### 3.1.2. Results with WGN

**Figure 4:**
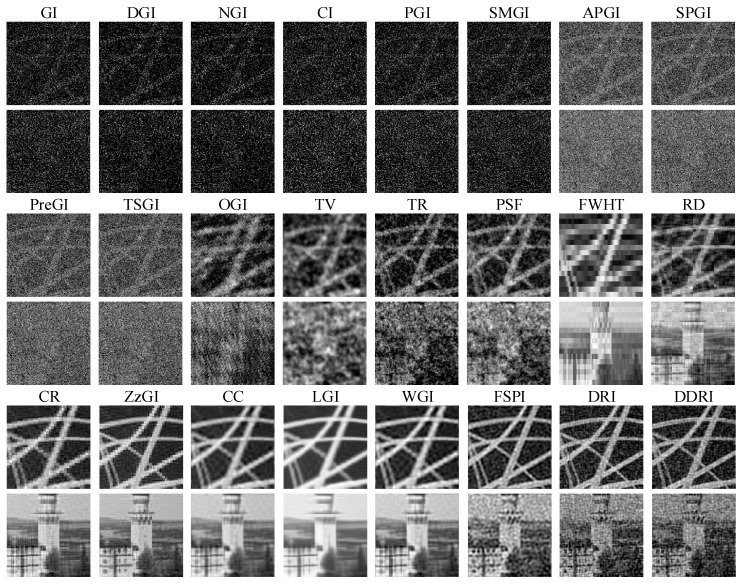
Simulation results of twenty-four methods with WGN. The noise level is 50.

**Figure 5:**
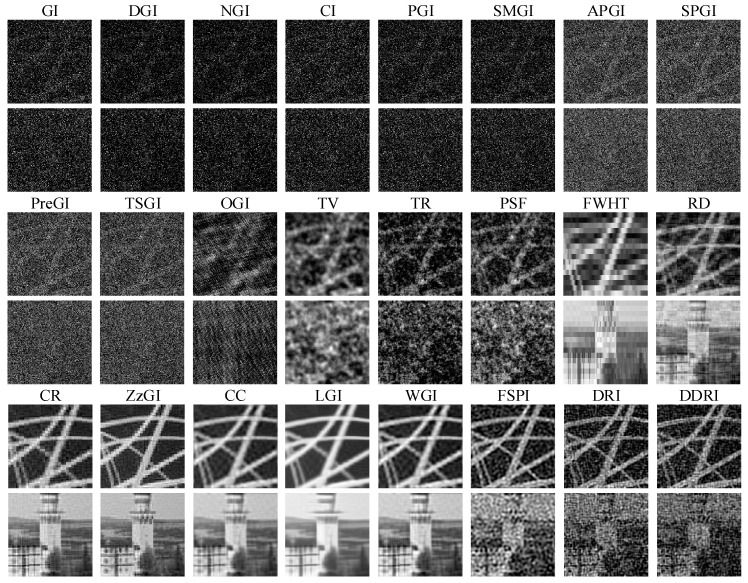
Simulation results of twenty-four methods with WGN. The noise level is 45.

Figure 4 and Figure 5 exhibit the recovered images with WGN, where the noise levels are respectively set as 50 and 45. The PSNR and RMSE of images corresponding to Figure 4 and Figure 5 are presented in Table 4 and Table 5, respectively. As shown in Figure 4 and Figure 5 and Table 4 and Table 5, WGN puts few influences on the recovered images of GI methods with Hadamard patterns. The image quality of Hadamard GI methods (e.g., CR, ZzGI, CC, etc.) shows little variation when the noise level changes. The image quality of FSPI decreases with the reducing of noise levels. DDRI and DRI are easily affected by the WGN. The image quality of the random pattern GI methods is poor even with the help of CS (OMP and TVAL3). Apart from the Hadamard GI methods, the antinoise capability of other methods (e.g., GI, DGI, APGI, DRI, etc.) is weak due to their special imaging theory.

### 3.2. Experimental Results and Analysis

**Figure 6:**
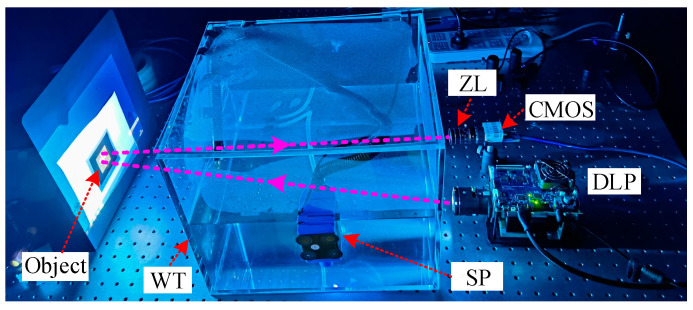
Experimental setup. ZL, zoom lens.

**Figure 7:**
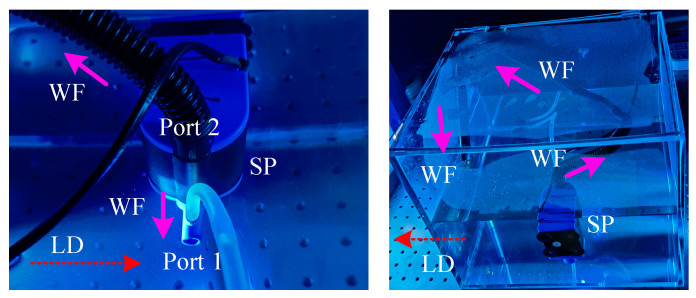
Relationship between the water flow (WF) and light direction (LD).

To verify the practical imaging performance of GI methods in underwater environment, a reflective UGI experiment setup was constructed, as shown in Figure 6. The setup includes a digital light projector (DLP), CMOS camera, water tank (WT), submersible pump (SP), and object. The DLP (F4710 LC, Fldiscovery, Jinhua, China), whose resolution is 1920 × 1080 pixels, is used to project the illumination patterns, such as the random and orthogonal patterns. The CMOS camera (Blackfly S BFS-U3-63S4C, 3027 × 2064 pixels, 60 fps) is used as the bucket detector. A zoom lens (HIKVISION, Hangzhou, China, focus length f=35 centimeter) is installed before the CMOS camera. The WT is made of the polymethyl methacrylate, whose refractive index and size are 1.49 and 45×30×30 cm, respectively. To create the underwater turbulence environment, an SP is mounted on the side wall of WT. The SP has two water-outlet ports, as shown in Figure 7. The directions of the water flow and light are plotted with the purple solid line and red dotted line, respectively. The test object is printed on a piece of white paper. In the experiments, the DLP and CMOS camera are controlled by a personal computer (PC, Intel Core i7-11,700 CPU, RAM 32 GB). The resolution of illumination patterns and the object image are all 128 × 128 pixels. Note that the images in Figure 6 and Figure 7 are captured by a mobile phone. During the mobile phone photographing, the submersible pump (SP) is turned down for safety. Consequently, the water is calm in Figure 6 and Figure 7. The ghost images generated by water in turbulence are shown in Section 3.2.3, where the SP is turned on to produce the simulated water turbulence.

**Figure 8:**
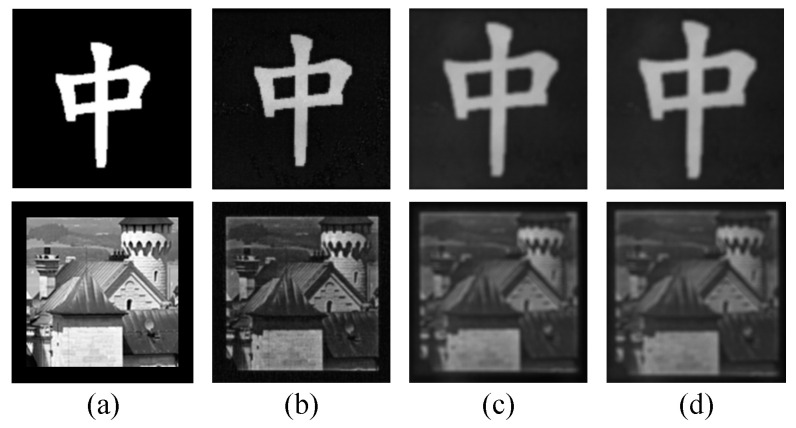
(**a**) is the original object image, (**b**–**d**) are respectively captured by the CMOS camera in the environment without water, with water, and with water and turbulence. (**b**–**d**) are cropped from the camera image and not scaled to (**a**).

Three groups of experiments are carried out: GI (1) without water, (2) with water, and (3) with water and turbulence. Note that all the experiments are finished in a darkroom, and the water in the experiments is the impure tap water (has some microparticles, not clear). Figure 8 shows the original image of the test object and the camera-captured experiment images. The test object contains the binary object “中” and grayscale object “house”.

#### 3.2.1. GI without Water

**Figure 9:**
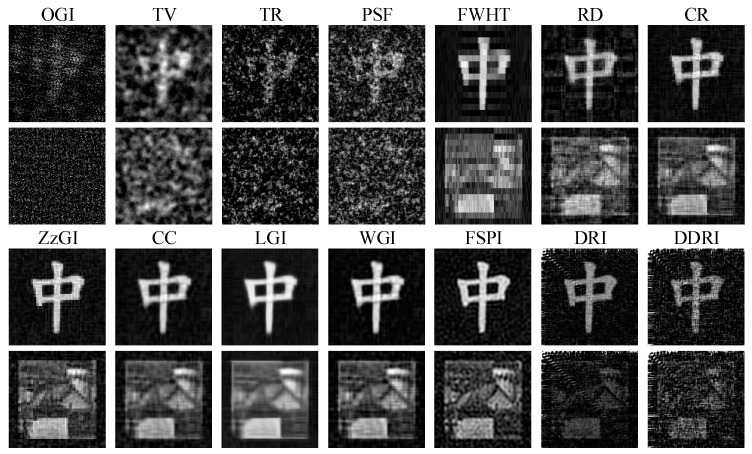
Experimental results of fourteen GI methods in the general environment (without WT).

In the first experiment, the WT was removed. Figure 9 shows the experimental results. As displayed in Figure 3, Figure 4 and Figure 5, the ten random pattern-based GI methods (e.g., GI, DGI, APGI, SPGI, etc.) show bad image quality in low measurement number conditions. Thus, these ten methods were dropped, and the other fourteen methods that have better imaging performance were used for experiments. As shown in Figure 9, the four random pattern-based CS GI methods (OGI, TV, TR, and PSF) cannot recover the clear images for objects “中” and “house”, while the Fourier, DCT, and other orthogonal pattern-based GI methods can restore the object images. However, the image quality of FWHT, DRI, and DDRI is low, where the images are contaminated by lots of noises. RD is a little better than FWHT, but the image quality is still not satisfied. The image quality of CR, ZzGI, CC, LGI, WGI, and FSPI is nearly the same from the direct vision. However, when we take a careful look at the images, WGI has the least noises and the best contrast.

Additionally, the PSNR and RMSE were used to evaluate the images in Figure 9. Note that only the PSNR and RMSE of the object “中” were calculated. The reason is that it is easy to create a reference image for the binary object “中”. It is hard to generate a reference image for the grayscale object “house” in the experiments. The PSNR and RMSE values corresponding to the images in Figure 9 are exhibited in Table 6. The PSNR and RMSE values of WGI are superior to the other seven methods, meaning that the image quality of WGI is the best.

#### 3.2.2. GI with Water

**Figure 10:**
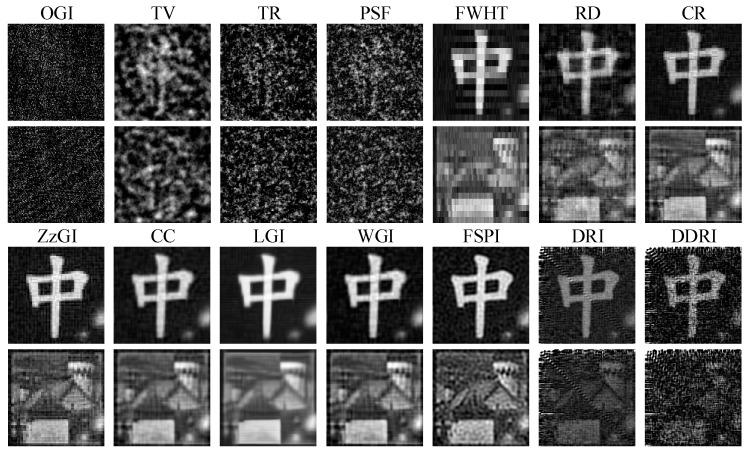
Experimental results of fourteen GI methods in the underwater environment (with WT, without turbulence).

**Figure 11:**
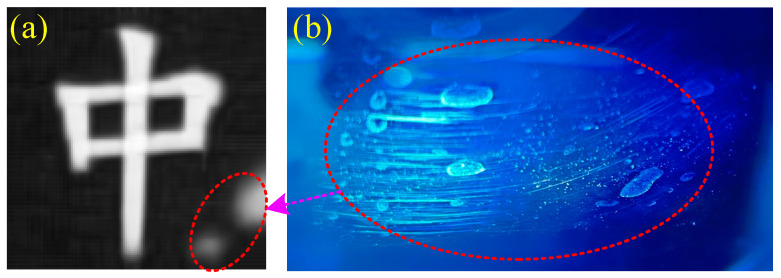
(**a**) UGI image restored by LGI, and (**b**) side wall image of the WT. The red dotted circles in (**a**,**b**) denote the possible relationship between the dirt stains and light spots.

In the second experiment, the WT was added, the SP was turned off, and the experimental setup is shown in Figure 6. Figure 10 shows the experimental results. The PSNR and RMSE of the object “中” corresponding to Figure 10 are presented in Table 7. Comparing Figure 10 and Table 7 with Figure 9 and Table 6, there are three major differences. Firstly, more noises appear in the recovered images after adding the WT. These are caused by the stray light from the experimental system. Here, the stray light is composed of three parts. One part is the back scattering light, and one part is the forward scattering light. These two stray lights are produced by the suspended microparticles in the impure tap water. The other part is the reflective light from the side wall of the WT. To reduce the influence of reflective lights, the WT was rotated by about 10 degrees around its central axis. Secondly, the objects in Figure 10 turn out to be larger than those in Figure 9. The reason is that the size of the illumination patterns was shrunk. Consequently, the objects of the restored images in Figure 10 become larger than those in Figure 9. Thirdly, two light spots were generated in the lower right corner of the reconstructed images. We checked the side wall of the WT and found that some dirt stains remained after washing the side wall of the WT. The dirt stains are shown in Figure 11, some of which may cause the unexpected light spots. These will be further investigated in future work.

As shown in Figure 10 and Table 7, the images of OGI, TV, TR, and PSF are unacceptable, and the images of FWHT, RD, DRI, and DDRI are not satisfactory even when the profiles of the objects are produced. The image quality of CC, LGI, WGI, and FSPI is better than the other ten methods, and WGI achieved the best imaging performance among the four methods.

#### 3.2.3. GI with Water and Turbulence

**Figure 12:**
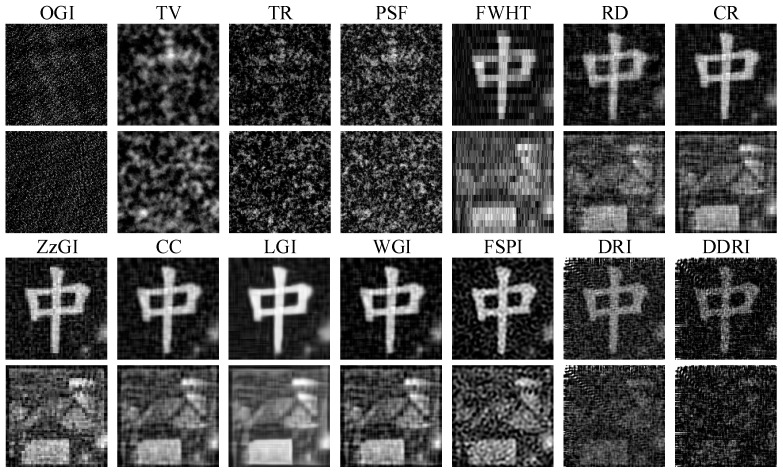
Experimental results of fourteen GI methods in the underwater environment (with WT, with turbulence).

In the third experiment, the experimental setup was the same as the one in Section 3.2.2, and the SP was turned on here. The experimental results are shown in Figure 12. Table 8 shows the PSNR and RMSE of the object “中” in Figure 12. Comparing Figure 12 with Figure 10, more noises are generated in Figure 12. Nonetheless, the image quality of CC, LGI, and WGI is acceptable for the binary object “中”. For the grayscale object “house”, the image quality of all the methods is low and not satisfied. Comparing Table 8 with Table 7, the PSNR values are decreased, and the RMSE values are increased in Table 8, indicating that the quality of images is decreased. Among the fourteen methods in Figure 12, the orthogonal pattern CS GI methods (CC, LGI, and WGI) demonstrate better antinoise capability and imaging performance.

## 4. Discussion

The simulation results in Figure 3, Figure 4 and Figure 5 and Table 3, Table 4 and Table 5 indicate that Hadamard GI methods have better antinoise performance than the other methods. In low measurement number conditions, without the presence of WGN, the Fourier, DCT, and other orthogonal pattern GI methods show better imaging performance than the random GI methods. Moreover, in the presence of WGN, the image quality of Hadamard pattern CS GI methods is superior to that of the random, Hadamard, and DCT patterns. In practical applications, a low measurement number can shorten the imaging time of GI. Consequently, the Hadamard CS GI methods have greater application prospects.

From the experimental results in Figure 9, Figure 10 and Figure 12 and Table 6, Table 7 and Table 8, we can find that the water and underwater turbulence reduce the image quality of GI methods. Compared with the images captured by the CMOS camera in Figure 8, GI can produce acceptable images for the binary object “中”. For the grayscale object “house”, the images of GI methods are worse than those obtained by the CMOS camera. However, the image quality of GI methods can be improved by increasing the measurement number. It should be noted that here the measurement number is about 3276 in the experiments. The measurement number can be increased to 10,000 or more to achieve a better imaging effect. Additionally, the CS GI methods (e.g., CC, LGI, and WGI) present excellent antinoise capability and imaging performance, which has great potential in practical applications.

As shown in Figure 9, Figure 10 and Figure 12, there is a diagonal artifact in the experimental images of DRI and DDRI methods, which is not presented in the numerical simulation images. The quality of the experimental DRI and DDRI images is much worse than the numerical results. The reasons may lie in the following two aspects. Firstly, DRI and DDRI use the predefined measurement and reconstruction matrices for the image restoration. Given a measurement matrix, only the corresponding reconstruction matrix and LIS are needed during the image restoration process. No special measures are taken to solve the external interference problem. Secondly, many complicated external interferences exist in practical experiments, such as the vibration noise, electron noise of the detector, environment light, etc. However, only the WGN is considered in the numerical simulations. These two aspects may lead to the image degradation of DRI and DDRI methods and the worse results compared with those of the numerical simulations.

Recently, deep learning has been applied in the GI area [36,68,69,70,71], with which excellent images can be recovered with few measurement numbers. However, deep learning GI requires lots of training data, which are not easy to acquire. Although the simulation methods can be used to create the training data [68,69], accurately simulating the complicated underwater environment (e.g., turbulence, various types of microparticles, turbidity, etc.) remains a challenge. Nevertheless, deep learning is a powerful and promising technique, and we will try to study the deep learning UGI schemes in future work. Additionally, we will try to develop the faster and better orthogonal pattern based UGI methods in future work.

Finally, from the numerical and experimental results, it can be found that the Hadamard pattern-based GI methods (e.g., CR, ZzGI, CC, LGI, etc.) are more suitable for imaging in the underwater environment than the other methods (e.g., OGI, FSPI, DRI, DDRI, etc.). The main reason is that a difference method is used to display the Hadamard pattern onto the SLP [35,49,50,51,55], and thus the corresponding light intensity is obtained by a difference calculation method. During the light intensity acquisition process, the noises can be reduced due to the difference calculation method. Consequently, Hadamard pattern-based GI methods demonstrate better imaging performance in numerical simulations and actual experiments owing to the fact that they have the LIS with fewer noises. Since the deep learning can dramatically improve the image quality of GI, the combination of the Hadamard patterns and deep learning technique is a perfect choice for UGI.

## 5. Conclusions

We have numerically and experimentally studied the imaging performance of twenty-four recently reported GI methods in the underwater environment. Three-group simulations and three-group practical experiments were conducted, respectively. The simulations and practical experiments were implemented in low measurement number conditions. The simulation results show that the image quality of the random pattern-based GI methods (without combining the CS) is very bad in low measurement number conditions. The experimental results show that the image quality of the random pattern-based GI methods is still very bad even with the help of CS. Both the simulations and practical experiments demonstrate that Hadamard pattern-based GI methods have strong antinoise capability and excellent imaging performance in the underwater environment. The Fourier pattern-based GI method (FSPI) also has good antinoise capability, but it cannot restore acceptable images in the underwater turbulence environment. The orthogonal pattern-based GI methods DRI and DDRI can achieve perfect images in the non-noise condition, but the image quality of these methods decreases dramatically in the noisy environment. In the future work, we will try to study the performance of GI in a water environment in which some oil is added.

## Figures and Tables

**Table 1:** Pattern types and methods used for simulations.

Pattern Type	GI Methods
Random	GI [13,14], DGI [46], NGI [47], OGI [52], TV [53], TR [56,57], SPGI [57], PSF [58], PGI [59], CI [62], PreGI [63], APGI [64], SMGI [65], and TSGI [67]
Orthogonal	WGI [35], FSPI [43], RD [48], CR [49], LGI [50], ZzGI [51], CC [55], DRI [60], DDRI [61], and FWHT [66]

**Table 2:** Simulation configurations.

Group	Adding WGN?	Noise Level
1	No	/
2	Yes	50
3	Yes	45

**Table 3:** PSNR and RMSE of images corresponding to Figure 3.

	OGI	TV	TR	PSF	FWHT	RD	CR
PSNR	14.86	13.45	20.26	18.63	14.70	14.88	17.74
	15.96	12.25	18.60	18.50	18.04	18.96	20.27
RMSE	0.18	0.21	0.10	0.12	0.18	0.18	0.13
	0.16	0.24	0.12	0.12	0.13	0.11	0.10
	**ZzGI**	**CC**	**LGI**	**WGI**	**FSPI**	**DRI**	**DDRI**
PSNR	18.73	19.07	18.79	19.31	20.96	23.29	22.66
	19.28	20.53	18.60	20.76	20.34	21.15	20.15
RMSE	0.12	0.12	0.11	0.11	0.09	0.07	0.07
	0.11	0.09	0.12	0.09	0.10	0.09	0.10

**Table 4:** PSNR and RMSE of images corresponding to Figure 4.

	OGI	TV	TR	PSF	FWHT	RD	CR
PSNR	11.53	12.93	11.91	13.64	14.70	14.91	17.70
	9.23	11.62	9.03	11.79	18.03	18.94	20.28
RMSE	0.27	0.23	0.25	0.21	0.18	0.18	0.13
	0.35	0.26	0.35	0.26	0.13	0.11	0.10
	**ZzGI**	**CC**	**LGI**	**WGI**	**FSPI**	**DRI**	**DDRI**
PSNR	18.75	19.06	18.77	19.30	18.15	17.36	16.50
	19.20	20.53	18.59	20.87	16.74	12.94	12.27
RMSE	0.12	0.11	0.12	0.11	0.12	0.14	0.15
	0.12	0.09	0.12	0.09	0.15	0.23	0.24

**Table 5:** PSNR and RMSE of images corresponding to Figure 5.

	OGI	TV	TR	PSF	FWHT	RD	CR
PSNR	9.37	12.50	9.73	11.11	14.71	14.93	17.67
	6.92	11.02	7.03	7.52	18.03	18.92	20.28
RMSE	0.34	0.24	0.33	0.29	0.18	0.18	0.13
	0.45	0.28	0.44	0.42	0.13	0.11	0.10
	**ZzGI**	**CC**	**LGI**	**WGI**	**FSPI**	**DRI**	**DDRI**
PSNR	18.75	19.05	18.80	19.29	16.16	13.99	13.78
	19.13	20.53	18.58	20.87	13.63	10.14	9.58
RMSE	0.12	0.11	0.11	0.11	0.16	0.20	0.20
	0.11	0.09	0.12	0.09	0.21	0.31	0.33

**Table 6:** PSNR and RMSE of images corresponding to the object “中” in Figure 9.

	FWHT	RD	CR	ZzGI	DRI
PSNR	15.29	15.32	16.79	16.93	13.73
RMSE	0.17	0.17	0.14	0.14	0.21
	**CC**	**LGI**	**WGI**	**FSPI**	**DDRI**
PSNR	17.30	17.72	18.71	17.78	12.17
RMSE	0.14	0.13	0.12	0.13	0.25

**Table 7:** PSNR and RMSE of images corresponding to the object “中” in Figure 10.

	FWHT	RD	CR	ZzGI	DRI
PSNR	14.41	14.06	14.97	15.80	11.14
RMSE	0.19	0.20	0.19	0.16	0.28
	**CC**	**LGI**	**WGI**	**FSPI**	**DDRI**
PSNR	15.98	16.53	17.08	16.40	10.62
RMSE	0.16	0.15	0.14	0.15	0.29

**Table 8:** PSNR and RMSE of images corresponding to the object “中” in Figure 12.

	FWHT	RD	CR	ZzGI	DRI
PSNR	13.38	13.13	14.11	14.40	10.78
RMSE	0.21	0.22	0.20	0.19	0.29
	**CC**	**LGI**	**WGI**	**FSPI**	**DDRI**
PSNR	14.93	15.83	15.55	14.39	9.99
RMSE	0.18	0.16	0.17	0.19	0.32

## Data Availability

Data underlying the results presented in this paper are not publicly available at this time but may be obtained from the authors upon reasonable request.
